# Perception and Prediction of Factors Influencing Carbon Price: Multisource, Spatiotemporal, Hierarchical Federated Learning Framework with Cross-Modal Feature Fusion

**DOI:** 10.3390/s25237274

**Published:** 2025-11-28

**Authors:** Peipei Wang, Xiaoping Zhou

**Affiliations:** 1 School of Economics and Management, Shanghai Open University, No. 288 Guoshun Road, Shanghai 200433, China; 2 School of Optical and Electronic Information, Suzhou City University, No. 1188 Wuzhong Avenue, Suzhou 215104, China

**Keywords:** carbon price, forecasting, feature selection, market influencing factors

## Abstract

To address the challenge of accurately predicting carbon price fluctuations, which are influenced by multiple factors, a multisource, spatiotemporal, federated learning framework with cross-modal feature fusion is proposed. Firstly, a three-level hierarchical federated learning network, consisting of perception clients, regional (edge) nodes, and a central server, is designed. The server incrementally aggregates the parameters generated by the local large model of the perception client through incremental data training, improving the efficiency of parameter aggregation in federated learning and avoiding the problem of network traffic data exposure. Secondly, a cross-modal, spatiotemporal, enhanced attention model is proposed. In order to extract the joint features of carbon price time series data and spatial correlation, spatiotemporal feature encoding is adopted. In order to share the semantic space of aligning market factors and carbon emission data in the embedding layer, cross-modal alignment is adopted. Finally, the experimental results demonstrate that the proposed framework can effectively predict carbon prices.

## 1. Introduction

With the increasing urgency of global climate change, carbon emissions and carbon trading have become critical environmental and economic concerns [1]. The development of carbon emission trading markets has steadily progressed, transforming carbon into a financial asset and providing an effective means to curb excessive emissions. Accurate prediction of carbon trading prices has become essential for informed decision making by governments, enterprises, and investors. However, carbon trading prices are characterized by strong volatility and instability and are significantly influenced by a variety of market factors. Reliable forecasting of carbon prices plays a crucial role in supporting evidence-based policymaking [2].

Existing studies have demonstrated that market factors affecting carbon prices include both economic variables and energy prices [3]. Economic variables encompass GDP growth rates, exchange rates, and capital market indicators, while energy prices include oil, coal, natural gas, and electricity prices [4,5]. Macroeconomic development shapes societal demand, thereby influencing carbon pricing dynamics [6,7]. For instance, to examine economic impacts, a spatial panel econometric model (SPEM) optimized with an ant colony algorithm was proposed to analyze how urban morphology and socioeconomic variables affect CO_2_ emissions [8]. Results indicated a 151% increase in emissions, exhibiting spatial clustering, underscoring the significant influence of urban form and economic development on emissions. Moreover, the effects of four types of economic policy uncertainty on carbon trading prices were analyzed using a nonlinear ARDL (autoregressive distributed lag) model and asymmetric causality tests [9,10].

To explore the combined effects of economic indicators and energy prices, the dynamic relationships between them and carbon prices were examined using a VAR-VEC (vector autoregressive–vector error correction) model [11], revealing long-term equilibrium relationships. Additionally, models such as ARMA-GARCH and grey correlation analysis were employed to explore the fluctuations in carbon prices and their potential links with energy and economic conditions [12]. Linear econometric models, such as Granger causality analysis, have also been used to study the relationships between carbon prices, stock markets, and macroeconomic variables [4,10]. However, such methods fail to capture the inherent nonlinearities and lag effects present in real-world multimodal market influences. Since different factors have varying degrees of influence on carbon prices, using irrelevant or weakly related variables may impair model performance. To address this, XGBoost (Extreme Gradient Boosting) has been introduced to capture complex nonlinear relationships more effectively [13,14], while simultaneously performing feature selection and dimensionality reduction to enhance model generalization and accuracy. The traditional centralized training methods mentioned above are prone to the following problems: The Internet of Things (IoT) devices generate different types of heterogeneous data from multiple sources, resulting in data silos and privacy security issues. How to effectively utilize data from IoT devices while ensuring data privacy and security has become an urgent need, hence the emergence of federated learning technology. In order to protect enterprise data privacy, a hierarchical federated learning framework is adopted to train a cross institutional carbon price prediction model. Multisource data fusion sensing technology is employed to achieve minute-level acquisition of carbon market factors, and the multisource data acquisition combined with the IoT equipment and real-time processing of edge computing. Lightweight edge nodes are deployed to process real-time data streams. The central node is responsible for global model aggregation and communication delay control.

To investigate the nonlinear causality and hysteresis between market factors and carbon prices, it is necessary to examine price dynamics across different temporal scales (daily, weekly, monthly, quarterly, and yearly). Decomposing raw carbon price data into homogeneous subsequences has become a standard approach to handling highly complex time series. Given the uncertainty and nonlinearity of carbon price data, ensemble empirical mode decomposition (EEMD) has been adopted to break down the original signal into multiple stable components and residuals, effectively suppressing high-frequency noise [15]. To capture long-term trends, complementary EEMD (CEEMD) has also been applied for a more refined decomposition of complex patterns [16]. Furthermore, CEEMDAN (Complete Ensemble Empirical Mode Decomposition with Adaptive Noise) has been used to smooth carbon price and volume data, improving data stationarity [17,18]. Nevertheless, due to the strong volatility and non-stationarity of carbon price signals, traditional models often face difficulties in parameter estimation and risk of overfitting. To overcome these issues, the adaptive chirp mode decomposition (ACMD) algorithm has shown advantages in extracting key features from multimodal signals with strong time-varying characteristics [19].

To better capture the temporal logic, regularity, and responsiveness of carbon price changes and their influencing factors, various prediction models have been proposed. For example, GARCH-based models have been used to analyze price volatility [20], while combinations of EMD and GARCH have been explored for enhanced forecasting [21]. However, these machine learning approaches often fail to fully resolve the issue of mode mixing during decomposition. In contrast, neural networks are capable of extracting useful features from large, fuzzy, and noisy datasets. Deep learning methods combined with wavelet transforms have been adopted to improve carbon price feature extraction and forecasting accuracy [22]. Hybrid models based on unstructured combinations have also been proposed to enhance predictive robustness [23]. For rapidly rising carbon prices, models that integrate GARCH with long short-term memory (LSTM) networks have been developed [24]. Other hybrid approaches combining LSTM with Light Gradient Boosting Machine (LGBM) have also demonstrated improved prediction performance [25,26,27,28]. Despite these advances, the nonlinear and unstable nature of carbon pricing continues to pose challenges. Temporal convolutional networks (TCNs), with their ability to extract features from complex time series, have recently emerged as promising alternatives [29].

In order to solve the problem of difficulty in accurately predicting changes in carbon prices due to multiple factors, a perception and prediction of carbon price influencing factors based on hierarchical federated learning and cross-modal spatiotemporal enhanced attention is proposed. Firstly, data hierarchical aggregation is achieved through multi-level nodes (such as edge devices, regional servers, and cloud centers) to optimize global model training efficiency while protecting local data privacy. Perception terminal devices (e.g., IoT sensors) perform local model training. Edge nodes (such as regional servers) aggregate multi-aware device parameters. Cloud center coordinates global model updates. Secondly, a cross-modal spatiotemporal enhanced attention perception network model is proposed. In order to extract the joint features of carbon price time series data and spatial correlation, spatiotemporal feature encoding is adopted. In order to share the semantic space of aligning market factors and carbon emission data in the embedding layer, cross-modal alignment is adopted. Thirdly, a dynamic attention mechanism is designed. The time attention mechanism is used to adaptively weigh the impacts of key time points. The spatial attention constructs a region correlation matrix through graph neural networks, highlighting the contribution of highly correlated nodes. Federated learning achieves collaborative training under multisource data privacy protection and combines time-delay dynamic weighting and multimodal fusion to improve carbon price prediction accuracy. Finally, experimental results confirm that the proposed method significantly improves the accuracy and robustness of carbon price forecasting.

## 2. Hierarchical Federated Learning Cross-Modal Spatiotemporal Enhanced Attention Construction

### 2.1. Hierarchical Federated Learning Framework

**Figure 1:**
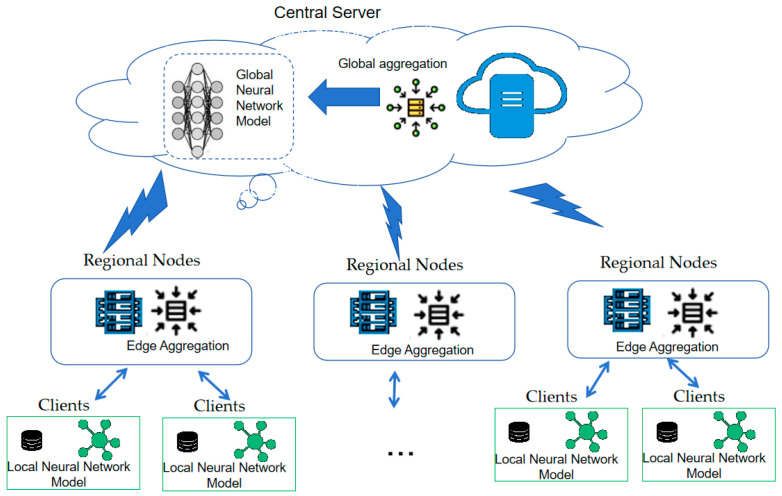
Hierarchical federated learning framework.

To clarify the conceptual hierarchy, the proposed model adopts a three-level federated architecture consisting of perception clients, regional (edge) nodes, and a central server. Each perception client (e.g., enterprise or institution) conducts local training using its own carbon trading and energy consumption data. The edge nodes aggregate parameters from multiple local clients, while the central server performs global aggregation and updates. This process preserves data locality and emulates hierarchical federated aggregation rather than centralized training. In our experiments, this communication structure is simulated in a controlled environment, with separate processes representing local, regional, and global updates. In order to protect enterprise data privacy, a hierarchical federated learning framework is adopted to train a cross institutional carbon price prediction model. Hierarchical federated learning reduces the central server’s workload through multi-level aggregation, making it well suited for environments with a large number of devices. In contrast, FedAvg follows a simple client–server architecture where all clients communicate directly with the central server and perform model updates through weighted averaging, without specific mechanisms to handle data heterogeneity or communication efficiency. FedProx adds a proximal term to FedAvg to improve stability but retains the same architecture. Hierarchical federated learning, on the other hand, offers enhanced privacy protection and energy efficiency [30,31]. The motivation for developing the hierarchical federated framework lies in addressing two main limitations of conventional federated learning: heterogeneous client resources and reliance on labeled data. The hierarchical federated learning framework is adopted to integrate heterogeneous data sources such as macroeconomic indicators, energy trading data, and policy texts through a dynamic feature alignment mechanism. The hierarchical Federated Learning uses a hierarchical aggregation mechanism to optimize communication efficiency and model performance. Its framework model is shown in Figure 1.

A three-tier architecture consisting of a central server, regional nodes and local perception clients is adopted. Assuming there are $N_{client}$ clients in the federated learning network, represented by $n_{client}$ = 1, 2, 3, …, $N_{client}$.

(1) Aware terminal device layer: Local training (such as IoT devices) is performed to generate model parameters. The multisource data fusion sensing technology is adopted to realize the minute-level data acquisition system of carbon price market factors, and the multisource data acquisition combined with the IoT equipment and real-time processing of edge computing.

The real-time data acquisition module integrates multimodal sensors, edge computing, and adaptive communication technology to dynamically monitor and analyze key parameters affecting market fluctuations.

(2) Edge node layer: The parameters of multiple perception terminal devices are aggregated to generate an edge model. Lightweight edge nodes are deployed to process real-time data streams. The central node is responsible for global model aggregation and communication delay control.

(3) Cloud server layer: All edge node parameters are aggregated to generate a global model. A horizontal–vertical hybrid federated architecture is adopted. The horizontal federated aggregation samples are used for homogeneous market data (such as carbon prices on various exchanges). The vertical federated alignment feature space is used for heterogeneous influencing factor data.

The goal of hierarchical federated learning: The objective function is the core optimization problem of federated learning, which achieves efficient distributed training through hierarchical aggregation. The global objective function minimizes both local loss and regularization terms while ensuring that original data remain on local devices. In each training round, local clients train models on their own data and send the updated parameters to regional servers. The regional models are then aggregated at the cloud center to form the global model, showing how the system operates in a small-scale experimental setting. To handle differences among clients, weighted aggregation is used when data sizes vary, and regularization is added to reduce the impact of non-independent and identically distributed data. These strategies help maintain stable convergence and efficient communication across heterogeneous clients. This study uses public carbon market data to test the proposed hierarchical federated learning framework as a prototype of a privacy-preserving system. In real applications, the same architecture can be applied to enterprises holding private data, where each client trains locally and shares only encrypted parameters through the hierarchical network. This ensures data privacy while enabling joint model learning across regions.

### 2.2. Cross-Modal Spatiotemporal Enhanced Attention Model

Enterprises or institutions hold private carbon trading data (such as historical carbon prices and energy consumption), which are locally trained through the model and only upload encrypted model parameters to regional nodes. Local data includes time series carbon prices, energy consumption, production data, and market impact factors dynamically injected into regional nodes through IoT. The federated spatiotemporal prediction network captures the interregional transmission effects of carbon prices. A multi-task learning architecture is adopted. The task of training short-term volatility prediction and long-term trend analysis is combined, and market factors are dynamically weighted through an attention mechanism.

**Figure 2:**
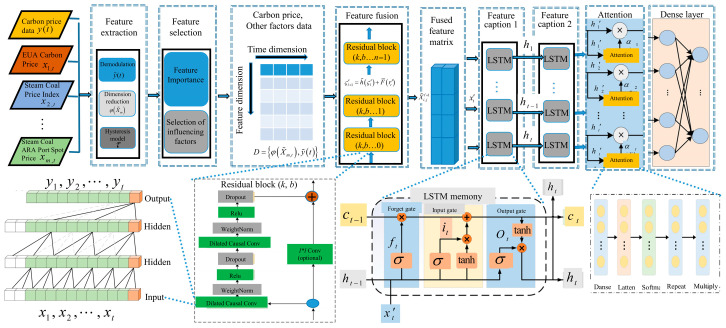
Cross-modal spatiotemporal enhanced attention mechanism.

To effectively capture the high-dimensional and dynamic interactions between carbon prices and multiple market influencing factors, a time–frequency lagged effect model is proposed. This model aims to fuse time-domain and frequency-domain features from both carbon price and its influencing variables, enabling a more nuanced understanding of temporal dependencies and market responses. The model architecture is designed to capture both short-term and long-term hysteresis effects and is capable of learning complex nonlinear relationships. In particular, an attention mechanism is incorporated to assign dynamic weights to different time steps, alleviating the issue of feature vanishing caused by increasing sequence lengths. This mechanism ensures that critical information is retained and emphasized in the modeling process. The overall architecture of the proposed model is illustrated in Figure 2.

The architecture comprises four key layers, each with distinct functionality:

- Horizontal Federation Aggregation Carbon Price Data: This layer accepts raw carbon prices and influencing factor time series. It utilizes time–frequency decomposition techniques for denoising and trend extraction. The goal is to perform quantitative analysis and eliminate the noise component of carbon price fluctuations, enabling the model to focus on stable trends and critical dynamics. Unified data descriptors are established during the feature engineering phase to ensure consistency across regional feature spaces.
- Vertical Federation Alignment Feature Space: To reduce dimensionality and model complexity, this layer computes the feature importance scores for each time–frequency component using mutual information, correlation metrics, or model-based ranking. Only the top-ranked features are passed on to subsequent layers. This step ensures that only the most relevant lagged features are retained for training. Privacy protection technology enables cross institutional data collaboration. In the gradient aggregation stage, weights are dynamically adjusted based on the contribution of features to model loss to optimize the fusion effect of market related features.
- Spatiotemporal Enhanced Attention Mechanism: The time attention mechanism is used to adaptively weigh the impacts of key time points. The spatial attention constructs a region correlation matrix through graph neural networks, highlighting the contribution of highly correlated nodes. The horizontal federated learning achieves collaborative training under multisource data privacy protection and combines time-delay dynamic weighting and multimodal fusion to improve carbon price prediction accuracy.
- Output Layer: The perception terminal sends the locally updated model parameters to the aggregation server. The server aggregates parameters such as weight matrix and bias vector.

## 3. Cross-Modal Horizontal–Vertical Hybrid Federation

### 3.1. Horizontal Federation Aggregation Carbon Price Data

The horizontal federated aggregation samples are used for homogeneous market data (such as carbon prices on various exchanges). Due to the strong volatility and instability of carbon prices, direct modeling of raw carbon price data tends to result in overfitting. Therefore, a model is needed to quantitatively analyze carbon prices and reveal their patterns, timeliness, and inherent logic. To improve the prediction accuracy of carbon prices, an econometric approach is adopted to capture the impact of market factors such as macroeconomic conditions, energy price fluctuations, market speculation, and alternative pricing mechanisms. Additionally, to address frequency inconsistencies across different time scales (daily, weekly, monthly, quarterly, and yearly), a decomposition technique is applied to the carbon price data. This enables the identification of various underlying trends, including seasonal effects, cyclical behaviors, and other periodic features embedded in the complex carbon price signal. The decomposition proceeds by first decomposing the price series, then solving an optimization problem to refine component separation.

#### 3.1.1. Construction of a Time–Frequency Matrix for Carbon Price Series

The input carbon price series undergoes time–frequency analysis, transforming the data from the time domain into the frequency domain to obtain its time–frequency representation. This process yields the distribution of signal energy across both time and frequency, laying the foundation for subsequent decomposition. Initially, background noise and outliers in y(t) were removed before analysis. During prediction, the weights $A_{n}(t)$ are adaptively learned to enhance both robustness and accuracy.

Unstable signals often consist of multiple sub-signals $y_{n}(t)$, each of which can be modeled by the following chirp-mode representation:

(1) $$y(t)=\sum_{n=1}^{N}y_{n}(t)+\varepsilon (t)=\sum_{n=1}^{N}A_{n}(t)\cos\left(2\pi \int_{0}^{t}B_{n}(\tau )d\tau +\theta_{n}\right)+\epsilon (t)$$

where y(t) is the observed carbon price data, N is the number of sub-signals, $A_{n}(t)>0$, $B_{n}(t)$, and $\theta_{n}$ represent the instantaneous amplitude, frequency, and initial phase of the sub-signals, respectively, and t∈(0,T].

The resulting time–frequency matrix derived from y(t) captures the primary spectral and temporal features of the signal. From this matrix, eigenvalues and eigenvectors are extracted to reveal the underlying structural patterns in the time–frequency domain. By leveraging these features and physical characteristics, modal components corresponding to various chirp patterns are identified and grouped.

To further isolate individual modal components from the original signal $y_{n}(t)$, a logarithmic transformation is applied, and Equation (1) is restructured as:

(2) $$\ln y(t)=\ln\left[\sum_{n=1}^{N}b_{n}(t)\cos\left(2\pi \int_{0}^{t}{\tilde{B}}_{n}(\tau )d\tau \right)+c_{n}(t)\sin\left(2\pi \int_{0}^{t}{\tilde{B}}_{n}(\tau )d\tau \right)+\ln\varepsilon (t)\right]$$

Here, $b_{n}(t)$ and $c_{n}(t)$ are demodulation operators, ${\tilde{B}}_{n}(t)$ denotes the chirp frequency functions. These operators are further defined as:

(3) $$\left\{\begin{array}{l} b_{n}(t)=A_{n}(t)\cos\{2\pi \int_{0}^{t}\left[B_{n}(\tau )-{\tilde{B}}_{n}(\tau )]dt+\theta_{n}\right\}\\ c_{n}(t)=-A_{n}(t)\cos\{2\pi \int_{0}^{t}\left[B_{n}(\tau )-{\tilde{B}}_{n}(\tau )]dt+\theta_{n}\right\} \end{array}\right.$$

The instantaneous amplitude is given by $A_{n}(t)=\sqrt{b_{n}^{2}(t)+c_{n}^{2}(t)}$. When $B_{n}(t)={\tilde{B}}_{n}(t)$, the frequency deviation vanishes, yielding periodic signals from which the subcomponents of the carbon price data can be estimated. The time–frequency matrix based on lny(t) then reveals periodic patterns and embedded regularities at different frequencies and time instances.

#### 3.1.2. Modal Feature Extraction and Iterative Decomposition Analysis

Based on the extracted chirp-mode features, the signal is decomposed iteratively into several chirped modal components using an adaptive algorithm. The decomposition parameters are dynamically tuned in accordance with the inherent characteristics of the data to ensure accurate separation of each modal component. The quality of decomposition is assessed by minimizing the mean square error, and the associated parameters are updated accordingly.

To solve the decomposition optimization problem under the assumption ${\tilde{B}}_{n}(t)=0$, the optimization task for the n-th sub-signal $\ln y_{n}(t)$ is defined as:

(4) $$\underset{b_{n}(t),c_{n}(t),y_{n}(t)}{min}\left\{{\left\|\ln b_{n}^{\prime \prime }(t)\right\|}_{2}^{2}+{\left\|\ln c_{n}^{\prime \prime }(t)\right\|}_{2}^{2}+\lambda {\left\|\ln y(t)-\ln y_{n}(t)\right\|}_{2}^{2}\right\}$$

where $b_{n}^{\prime \prime }(t)$ is the second derivative of $b_{n}(t)$, $c_{n}^{\prime \prime }(t)$ is the second derivative of $c_{n}(t)$, $\|\cdot {\|}_{2}$ denotes the $L^{2}$-norm, and λ is the penalty factor. Let N denote the number of time samples; the discrete form of Equation (4) becomes:

(5) $$\underset{\Theta \left(t\right),\theta_{n}\left(t\right)}{min}\left\{{\left\|\ln\left(\Theta \left(t\right)g_{n}\left(t\right)\right)\right\|\;\;}_{2}^{2}+\lambda {\left\|\ln y\left(t\right)-\ln\left(\theta_{n}\left(t\right)g_{n}\left(t\right)\right)\right\|}_{2}^{2}\right\}$$

Here, $\Theta \in {\mathbb{R}}^{(2N-4)\times 2N}$ is a block-diagonal matrix constructed from the second-order finite difference operator Υ, which enforces smoothness: Θ=blockdiag(Υ,Υ); $F_{n}\in {\mathbb{R}}^{N\times 2N}$ is the design matrix defined as: $F_{n}=\left[E_{n}\;S_{n}\right]$, with $E_{n}=\mathrm{diag}\left(\cos\theta_{n}(t_{0}),\ldots ,\cos\theta_{n}(t_{N-1})\right)$ and $S_{n}=\mathrm{diag}\left(\sin\theta_{n}(t_{0}),\ldots ,\sin\theta_{n}(t_{N-1})\right)$.

During the iterative decomposition, each extracted modal component is subtracted from the original signal before proceeding to extract the next one. This ensures that each chirped mode contains only one dominant physical pattern. The iteration terminates when the residual of the signal satisfies a predefined threshold.

The reconstructed signal then consists of the sum of all modal components and the final residual:

(6) $$\ln\tilde{y}(t)=\ln\sum_{n=1}^{N}{\tilde{y}}_{n}(t)+\ln r_{n}(t)$$

where $\ln{\tilde{y}}_{n}(t)$ is the estimated *n*-th modal subcomponent, and $\ln r_{n}(t)$ is the residual. Based on this decomposition model of Equation (6), the temporal variation laws of amplitude, frequency, and phase of the carbon price signal can be quantitatively characterized.

### 3.2. Vertical Federation Alignment Feature Space

A vertical federated alignment feature space is used to handle heterogeneous influencing factor data. Spatiotemporal alignment and feature aggregation of multisource market influencing factors are implemented. Because market factors exhibit multisource heterogeneity, high correlation, and hysteresis, predicting carbon prices is inherently challenging. It is therefore necessary to analyze how these market factors influence carbon price fluctuations and reduce the complexity of input data through dimensionality reduction.

First, the data for M market influencing factors over T time steps is organized into a T×M matrix X, where each column corresponds to one influencing factor:

(7) $$X=\left[\begin{array}{cccc} x_{1,1} & x_{1,2} & \ldots & x_{1,M}\\ x_{2,1} & x_{2,2} & \ldots & x_{2,M}\\ \vdots & \vdots & \ddots & \vdots \\ x_{T,1} & x_{T,2} & \ldots & x_{T,M} \end{array}\right]$$

Let $x_{t,m}$ represent the value of the m-th influencing factor at time t. Each column $X_{m}$ is mapped to a high-dimensional feature space using a nonlinear transformation φ. The mean of the transformed feature is:

(8) $$\mu_{m}=\frac{1}{M}\sum_{m=1}^{M}\varphi (X_{m})$$

Each feature is then normalized:

(9) $${\tilde{X}}_{m}=\frac{\varphi (X_{m})-\mu_{m}}{\sigma_{m}}$$

where $\sigma_{m}$ is the standard deviation of $\varphi (X_{m})$, i.e.,

(10) $$\sigma_{m}=\sqrt{\frac{1}{M}\sum_{m=1}^{M}{\left[\varphi (X_{m})-\mu_{m}\right]}^{2}}$$

The kernel matrix K is constructed using the kernel function of a multi-layer perceptron. The elements of $K\in {\mathbb{R}}^{M\times M}$ are computed as:

(11) $$K_{m,m^{\prime }}=\exp\left(\frac{\left|\varphi \left({\tilde{X}}_{m}\right)-\varphi \left({\tilde{X}}_{m^{\prime }}\right)\right|}{-\delta^{2}}\right)$$

where δ is the bandwidth of the Gaussian kernel, $1\leq m,m^{\prime }\leq M$.

To center the kernel matrix, the normalized (standard) kernel matrix $\hat{K}$ is computed as:

(12) $$\hat{K}=K-I_{1/M}K-KI_{1/M}+I_{1/M}KI_{1/M}$$

where $I_{1/M}\in {\mathbb{R}}^{M\times M}$ is a matrix with all elements equal to 1/M.

To address the multicollinearity problem caused by strong correlations among market influencing factors, principal component analysis (PCA) is employed. By extracting the principal components, redundant information is effectively removed, allowing for the identification of the most relevant features.

The eigenvalues and eigenvectors of the covariance matrix of the market influencing factors are computed to quantify the correlation structure between variables. The covariance matrix Σ of the centered kernel matrix is defined as:

(13) $$\Sigma =\frac{1}{M}\sum_{m=1}^{M}{\hat{K}}_{m}{\hat{K}}_{m}^{T}$$

where ${\hat{K}}_{m}$ is the m-th column of the centered kernel matrix $\hat{K}$, and $\Sigma \in {\mathbb{R}}^{M\times M}$.

To perform PCA, the singular value decomposition (SVD) of the covariance matrix is calculated:

(14) $$\Sigma =U\Sigma^{\prime }V^{*}$$

where $\Sigma^{\prime }$ is a diagonal matrix containing the eigenvalues of Σ, $U\in {\mathbb{R}}^{M\times M}$ is the matrix of left singular vectors (eigenvectors), $V^{*}\in {\mathbb{R}}^{M\times M}$ is the conjugate transpose of the right singular vector matrix.

The eigenvectors in U are ordered according to descending eigenvalues:

(15) $$U^{\prime }=[u_{1},u_{2},\ldots ,u_{\bar{m}},\ldots ,u_{\bar{M}}]$$

where $u_{\bar{m}}$ is the eigenvector corresponding to the $\bar{m}$-th largest eigenvalue of the covariance matrix Σ. These eigenvectors define the directions of maximum variance in the transformed feature space.

The eigenvectors are sorted in descending order of their corresponding eigenvalues, and the top principal components are selected. The larger the eigenvalue, the greater the variance of the data along the associated eigenvector direction, and thus, the more information it retains.

The original data is projected onto the selected principal components, effectively transforming it into a new low-dimensional feature space. This feature space is defined by the selected eigenvectors. By performing matrix multiplication, the transformed data is obtained by projecting the standardized feature data onto the reduced eigenspace.

The reduced feature matrix $z_{m}$ is calculated as:

(16) $$z_{m}=U^{\prime \;T}\varphi (X_{m})$$

This dimensionality reduction process helps eliminate multicollinearity present in the original market influencing factor dataset. As a result, only the most strongly correlated and informative features are retained for subsequent modeling.

### 3.3. Modeling of Hysteresis Effects and Automated Feature Selection

#### 3.3.1. Hysteresis Effect Identification and Modeling

To model the hysteresis effects in market influencing factors, lagged versions of each variable are treated as candidate features. Each variable is analyzed individually to evaluate its time-delayed impact on carbon price fluctuations. Specifically, the hysteresis period (i.e., time lag) at which a given variable exhibits the strongest correlation with carbon price is identified.

For each factor, correlation coefficients between its lagged versions (from 0 to 12 months) and the carbon price are calculated. The lag order with the highest correlation is selected and further verified using Granger causality testing to assess statistical significance. Three approaches are adopted: time-shifted cross-correlation analysis, Granger causality test, and lagged regression modeling.

##### Time-Shifted Cross-Correlation Analysis

The objective of this method is to determine the correlation coefficient between different hysteresis orders (e.g., 0 to 12 months) of market influencing factors and the carbon price. The time lag corresponding to the maximum correlation is then identified.

Given the standardized time series $\varphi ({\tilde{X}}_{m,t})$ for the m-th influencing factor and the logarithmic carbon price series $\ln\tilde{y}(t)$, index restoration is performed as $\tilde{y}(t)=\exp(\ln\tilde{y}(t))$. The time-shifted cross-correlation function is defined as:

(17) $$R_{m,\tau }\left(\varphi ({\tilde{X}}_{m,t-\tau }),\tilde{y}(t)\right)=\sum_{t=1}^{T-\tau }\varphi ({\tilde{X}}_{m,t-\tau })\cdot \tilde{y}(t)$$

where τ denotes the hysteresis order (in months), $\varphi ({\tilde{X}}_{m,t-\tau })$ is the lagged time series of the m-th influencing factor, and $\tilde{y}(t)$ is the carbon price at time t−τ. For high-frequency data (e.g., daily), a rolling window analysis is used to dynamically assess the variation in optimal lag order τ.

To evaluate nonlinear correlations, mutual information coefficient (MIC) is computed between the lagged feature $\varphi ({\tilde{X}}_{m,t})$ and the carbon price $\tilde{y}(t)$ across different delay orders τ. The dataset $D=\{\varphi ({\tilde{X}}_{m,t}),\tilde{y}(t)\}$ is used to compute mutual information:

(18) $$\Phi {\left(D\right)}_{\varphi \left({\tilde{X}}_{m,t}\right),\tilde{y}\left(t\right)}=\underset{m\times \tau <\left(M+1\right)\times \bar{T}\;}{\max}\frac{I\left(\varphi \left({\tilde{X}}_{m,t-\tau }\right);\tilde{y}\left(t\right)\left|m\times \tau \right.\right)}{\log_{2}\min\left\{m,\tau \right\}}$$

Here, $I(\varphi ({\tilde{X}}_{m,t}),\tilde{y}(t),D,T,\tau )$ denotes the mutual information between $\varphi ({\tilde{X}}_{m,t})$ and $\tilde{y}(t)$ over a grid of T rows and M+1 columns.

A grid search is then performed over all possible $\varphi ({\tilde{X}}_{m,t})\times \tilde{y}(t)$ 2D grids to identify the configuration that maximizes the standardized mutual information. The *MIC(D)* of the dataset is assigned to the highest point on the resulting feature surface:

(19) $$MIC\left(D\right)=\underset{\varphi \left({\tilde{X}}_{m,t}\right)*\tilde{y}\left(t\right)<\bar{B}}{\max}\Phi {\left(D\right)}_{\varphi \left({\tilde{X}}_{m,t}\right),\tilde{y}\left(t\right)}$$

where * indicates the grid product and $\bar{B}$ is a threshold for the product.

If $\varphi ({\tilde{X}}_{m,t})$ and $\tilde{y}(t)$ are statistically independent under all hysteresis orders, the mutual information is zero. Conversely, maximum MIC implies strong nonlinear dependence.

The algorithm is then used to rank the influence of each lagged variable based on its correlation with carbon price under different hysteresis orders. The direction of hysteresis is inferred from the sign of the correlation coefficient. The variable and lag with the highest MIC or correlation are retained for prediction.

##### Granger Causality Test

To verify whether the lagged variable $\varphi ({\tilde{X}}_{m,t-\tau })$ provides predictive power for $\tilde{y}(t)$, the Granger causality test statistic is calculated as:

(20) $$GC=\frac{\left(RSS_{0}-RSS_{1}\right)/\tau_{1}}{RSS_{1}/\left(\bar{T}-2\tau_{2}-1\right)}$$

where $\tau_{1}$ is the lag order of $\varphi ({\tilde{X}}_{m,t-\tau })$, $\tau_{2}$ is the lag order of $\tilde{y}(t)$, ${\mathrm{RSS}}_{0}$ and ${\mathrm{RSS}}_{1}$ are the residual sum of squares of the restricted and unrestricted models, respectively. If *GC* exceeds the critical value, the lagged variable is considered to have significant predictive effect.

##### Lagged Variable Regression Modeling

Once the optimal hysteresis order $\tau^{*}$ for each market influencing factor is determined through time-shifted cross-correlation, mutual information, or Granger causality analysis, the corresponding lagged features $\varphi ({\tilde{X}}_{m,t-\tau^{*}})$ are incorporated into the predictive regression model.

These lagged variables serve as nonlinear explanatory factors that reflect the delayed impact of market signals on carbon price dynamics. Due to the complexity and potential multicollinearity of such lagged variables, traditional linear regression models may not fully capture their effects. Therefore, tree-based ensemble learning methods, particularly gradient boosting regression models, are adopted to model these nonlinear dependencies.

#### 3.3.2. Automated Selection of Influential Features

Since the influence of different market factors on the carbon price varies in intensity and temporal delay, building an accurate prediction model requires the elimination of redundant and irrelevant features. In this study, we adopt an automatic feature selection mechanism to identify the most significant variables for carbon price prediction. These selected features not only provide relevant information but also encapsulate hysteresis effects embedded in the complex temporal dynamics of the original dataset.

To enhance the generalization capability and predictive accuracy of the model, we calculate the correlation coefficient between each influencing factor and the carbon price across different hysteresis orders. The maximum correlation value for each variable is then extracted and used to determine the optimal lag configuration.

Once the significant features are identified, an ensemble learning framework is applied. In this framework, weak learners (decision trees) are iteratively added to minimize the prediction error. At each stage, the residuals from the previous iteration are used to train the next learner. After a sufficient number of iterations, the outputs of all weak learners are aggregated to produce the final prediction result.

The prediction function of the ensemble model is expressed as:

(21) $$\hat{y}(t)=\sum_{k=1}^{\bar{K}}f_{k}\left(\varphi \left({\tilde{X}}_{m,t-\tau }\right),\tilde{y}(t)\right),\;f_{k}\in F$$

where $\hat{y}(t)$ is the predicted carbon price at time t, $f_{k}$ denotes the k-th regression tree, $\bar{K}$ is the number of trees in the ensemble, and F is the function space of all possible CART sets.

The objective function for the model training process is defined as:

(22) $$\mathrm{Obj}=\sum_{t=1}^{T}l\left(y(t),\hat{y}(t)\right)+\sum_{k}\Omega (f_{k})+\bar{C}$$

where l(⋅) is the loss function, $\Omega (f_{k})$ is the regularization term that penalizes model complexity, and $\bar{C}$ is a constant.

The regularization term is defined as:

(23) $$\Omega (f_{k})=\gamma \zeta +\frac{1}{2}\xi \|w{\|}^{2}$$

where γ is the regularization coefficient, ζ is the number of leaves in the k-th tree, w is the weight vector of the leaves, and ξ is the weight penalty parameter.

To simplify the objective function, a second-order Taylor expansion is applied:

(24) $${\mathrm{Obj}}^{\left(k\right)}\approx \sum_{t=1}^{\bar{T}}\left[l(\hat{y}(t),\tilde{y}(t))+g_{t}f_{k}(t)+\frac{1}{2}h_{t}f_{k}^{2}(t)\right]+\Omega (f_{k})$$

where $g_{t}$ and $h_{t}$ are the first and second derivatives of the loss function:

(25) $$g_{i}={\left.\frac{\partial l(z_{i},z_{i}^{*})}{\partial z_{i}^{*}}\right|}_{z_{i}^{*}={\hat{y}}^{\left(t-1\right)}}$$

(26) $$h_{i}={\left.\frac{\partial^{2}l(z_{i},z_{i}^{*})}{\partial {z_{i}^{*}}^{2}}\right|}_{z_{i}^{*}={\hat{y}}^{\left(t-1\right)}}$$

The additive model assumption implies that each newly trained function is added to the existing ensemble without altering previous trees. The simplified objective function becomes:

(27) $${\mathrm{Obj}}^{\left(k\right)}=\sum_{t=1}^{\bar{T}}\left[g_{t}f_{k}(t)+\frac{1}{2}h_{t}f_{k}^{2}(t)\right]+\gamma \zeta +\frac{1}{2}\xi w^{2}$$

Given the complexity of tuning γ and ξ in Equation (27), Bayesian optimization is used to adaptively select optimal hyperparameters before training. This ensures better generalization and improved learning efficiency. The hyperparameter optimization problem is:

(28) $$\arg\min_{\gamma ,\xi }\mathrm{Obj}(\gamma ,\xi )$$

Let $q^{*}=\{\gamma^{*},\xi^{*}\}$ be the optimal solution. The steps for Bayesian optimization are as follows:

- Step 1: Initialize candidate set $Q_{\mathrm{init}}=\{q_{0},\ldots ,q_{i-1}\}$.
- Step 2: Evaluate function value $\varpi (Q_{\mathrm{init}})$, and compute initial evaluation set $\Xi_{0}=\{Q_{\mathrm{init}},\varpi (Q_{\mathrm{init}})\}$.
- Step 3: Build the surrogate model from $\Xi_{0}$.
- Step 4: Choose the next evaluation point by minimizing the acquisition function:
  (29) $$q_{i-1}=\arg\min_{q}\;\alpha (q|\Xi_{i-1})$$
- Step 5: Add the new evaluation $\varpi (q_{i-1})$ to the evaluation set:
  (30) $$\Xi_{i}=\Xi_{i-1}\tilde{U}\left\{q_{i-1},\varpi (q_{i-1})\right\}$$
- Step 6: Repeat until convergence and return the best hyperparameter combination $q^{*}=\{\gamma^{*},\xi^{*}\}$.

It is important to clarify the implementation scope of the proposed framework. In the current study, we focus on vertical feature federation and time series modeling within a unified prediction network. While a hierarchical federated architecture (client ⟶ edge ⟶ cloud) is introduced conceptually to illustrate the potential deployment framework, the present experiments do not implement parameter-level hierarchical model aggregation or heterogeneous small-model fusion across nodes. Instead, the experiments simulate the communication structure in a controlled environment to evaluate the model’s prediction performance using public carbon market datasets. The exploration of hierarchical model weight aggregation and small model–large model fusion is identified as an important direction for future work, particularly for real-world deployments involving privacy-sensitive enterprise data.

## 4. Spatiotemporal Enhanced Attention Mechanism

The hierarchical federated learning network adopts a multi-task learning architecture to capture the transmission effect of carbon prices between regions. The task of training short-term volatility prediction and long-term trend analysis is combined, and market factors are dynamically weighted through an attention mechanism.

### 4.1. Multiscale Spatiotemporal Modeling

The feature fusion network layer is designed to effectively capture time-varying patterns of carbon price and multiple market influencing factors across different temporal scales. To achieve this, the model incorporates stacked dilated convolution layers, pooling layers, and fully connected layers, enabling the fusion of both short- and long-term dependencies in multivariate time series data.

**Figure 3:**
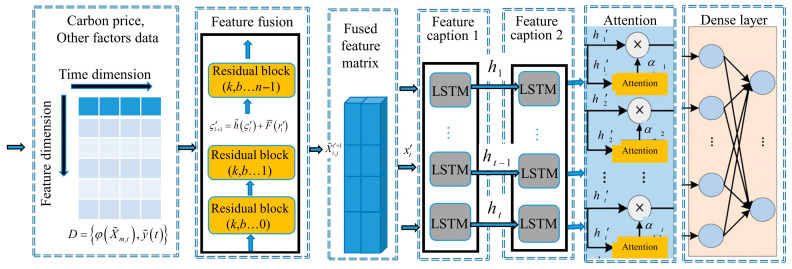
Multiscale spatiotemporal modeling framework.

In this architecture, the dilated convolution (also known as hole convolution) plays a key role in extracting features across various time scales without increasing computational cost. By adjusting the dilation (void) rate, the receptive field of the convolutional filters is expanded, allowing the network to capture patterns from wider temporal contexts. The multiscale spatiotemporal model is illustrated in Figure 3.

The model receives inputs $\left\{\varphi \left({\tilde{X}}_{m,t}\right)⊕\tilde{y}\left(t\right)\right\}$ and processes them via causal convolutions that respect the time order. To capture different time-scale dependencies, dilated convolutions are applied. The hole (dilated) convolution operation is formulated as:

(31) F¯X˜m,t,y˜t=ReLUCrossAttentionh¯m,t,y˜t+b¯, h¯m,t=∑k=0K′−1f¯kφX˜m,t−d′⋅k

where ${\bar{h}}_{m,t}$ denotes the extracted multiscale feature representation, f(k) is the k-th element of the convolution kernel, and *d*’ is the dilation factor. ${\tilde{X}}_{m}$ represents the input sequence of the m-th modality, and t is the time index. ReLU(⋅) denotes the activation function, and $\bar{b}$ is the bias term. CrossAttention(⋅) represents the cross-attention fusion mechanism, which receives the feature maps from dilated convolutions with different dilation rates. These multiscale tensors are concatenated along the specified dimension to connect features across multiple temporal resolutions. After splicing and fusion, the resulting feature tensor has $\bar{T}$ rows and M+1 columns.

Residual connections are introduced to form deeper architectures and mitigate gradient vanishing. Each residual block consists of a skip connection and a convolutional transformation:

(32) $$\zeta_{l+1}^{\prime }=\tilde{h}(\zeta_{l}^{\prime })+\bar{F}(r_{t}^{\prime }),$$

where $\tilde{h}(\zeta_{l}^{\prime })=W_{l}^{\prime }\zeta_{l}^{\prime }$ represents a 1 × 1convolution-based direct mapping.

To optimize feature fusion network hyperparameters (e.g., filter size, depth, learning rate), we use the Sparrow Search Algorithm (SSA), a bio-inspired metaheuristic optimization technique. Each sparrow represents a candidate hyperparameter vector, encoded in a position matrix:

(33) $$\tilde{X}=\left[\begin{array}{ccc} {\tilde{X}}_{1,1} & \ldots & {\tilde{X}}_{1,d}\\ \vdots & \ddots & \vdots \\ {\tilde{X}}_{n^{\prime },1} & \ldots & {\tilde{X}}_{n^{\prime },d} \end{array}\right],$$

where ${\tilde{X}}_{n^{\prime },d}$ denotes the *d*-th dimension of the *n*′-th sparrow. *n*′ represents the number of sparrows, and *d* represents the dimension of the variable to be optimized.

The fitness of each individual is calculated based on model performance:

(34) $$F_{\tilde{X}}=\left[\begin{array}{c} \tilde{f}({\tilde{X}}_{1,:})\\ \vdots \\ \tilde{f}({\tilde{X}}_{n^{\prime },:}) \end{array}\right].$$

where the value of each row in ${\tilde{F}}_{\tilde{X}}$ indicates the fitness value of the individual. SSA includes distinct update rules for producers, scroungers, and sentinels. Producers update their positions as:

(35) $${\tilde{X}}_{i,j}^{t+1}=\left\{\begin{array}{ll} {\tilde{X}}_{i,j}^{t}\cdot \exp\left(\frac{-i}{\alpha \cdot iter_{\max}}\right), & R_{2}<ST,\\ {\tilde{X}}_{i,j}^{t}+Q^{\prime }/L, & R_{2}\geq ST \end{array}\right.$$

where $t^{\prime }$ denotes the current iteration, j=1,2,…,d. ${\tilde{X}}_{i,j}^{t^{\prime }}$ denotes the value of the j-th dimension of the first sparrow at iteration $t^{\prime }$. $iter_{max}$ is the constant with the highest number of iterations. $\alpha^{\prime }\in \left(0,1\right)$ is a random number. $R_{2}\in [0,1]$ and ST∈[0.5,1.0] stand for the alarm value and the safety threshold, respectively. $Q^{\prime }$ is a random number obeying the normal distribution. $L^{\prime }$ denotes a 1×d matrix, where each element is 1. When $R_{2}<ST$, the producer enters the wide search mode.

The position update rule for vigilant scroungers is:

(36) $${\tilde{X}}_{i,j}^{t+1}=\left\{\begin{array}{ll} Q^{\prime }\cdot \exp\left(\frac{{\tilde{X}}_{\mathrm{worst}}^{t}-{\tilde{X}}_{i,j}^{t}}{i^{2}}\right), & i>\frac{n^{\prime }}{2}\;\;\;.\\ {\tilde{X}}_{p}^{t+1}+|{\tilde{X}}_{i,j}^{t}-{\tilde{X}}_{p}^{t+1}|\cdot A^{+}\cdot L, & \mathrm{otherwise} \end{array}\right.$$

where ${\tilde{X}}_{P^{\prime }}$ is the best position occupied by the producer. ${\tilde{X}}_{\mathrm{worst}}$ denotes the current global worst position. $A^{\prime }$ denotes the matrix of 1×d, which each element is randomly assigned a value of 1 or −1, and $A^{+}={\left(A^{\prime }\right)}^{T}{\left(\left(A^{\prime }\right){\left(A^{\prime }\right)}^{T}\right)}^{-1}\;$. When $i>n^{\prime }/2$, it indicates that the i-th gleaner with the worse fitness value is most likely to starve. Danger-aware sparrows (10–20\% of the population) have specialized update rules:

(37) $${\tilde{X}}_{i,j}^{t+1}=\left\{\begin{array}{ll} {\tilde{X}}_{\mathrm{best}}^{t}+\beta \cdot |{\tilde{X}}_{i,j}^{t}-{\tilde{X}}_{\mathrm{best}}^{t}|, & f_{i}>f_{g}\\ {\tilde{X}}_{i,j}^{t}+K^{\prime }\cdot \frac{{\tilde{X}}_{i,j}^{t}-{\tilde{X}}_{\mathrm{worst}}^{t}}{(f_{i}-f_{w})+\varepsilon^{\prime }}, & f_{i}=f_{g} \end{array},\right.$$

where ${\tilde{X}}_{best}$ is the current global optimal position. The step control parameter β is a normal distribution with mean 0 and variance 1. $\delta \in \left[-1,1\right]$ is a random number. ${f_{i}}^{\prime }$ is the current fitness value of the sparrow. ${f_{g}}^{\prime }$ and ${f_{w}}^{\prime }$ are the current global optimal and worst fitness values, respectively. $\varepsilon^{\prime }$ is the smallest constant to avoid division by zero error. ${f_{i}}^{\prime }>{f_{g}}^{\prime }$ indicates that the sparrow is at the edge of the flock for simplicity. ${\tilde{X}}_{best}$ represents the position of the center of the population, around which it is safe.

Through SSA, the feature fusion network model achieves a more effective configuration of its architecture and learning dynamics, leading to enhanced accuracy in capturing complex patterns in carbon price fluctuations.

After the multiscale temporal features are extracted, we employ a gated recurrent structure to capture long-range dependencies across the time series. This recurrent mechanism is specifically designed to mitigate issues such as vanishing gradients, thereby enabling the model to effectively learn long-term patterns in carbon price dynamics. The internal operations of the recurrent unit at time step t are defined by the following recursive update equations:

(38) $$\left(\begin{array}{c} f_{t}\\ i_{t}\\ o_{t}\\ {\bar{C}}_{t} \end{array}\right)=\left(\begin{array}{c} \sigma \\ \sigma \\ \sigma \\ \tanh \end{array}\right)\left(\overset{⌢}{W}\cdot \left[h_{t-1},{x^{\prime }}_{t}\right]+\overset{⌢}{b}\right)$$

where $W^{\prime \prime }$ is the merged weight matrix, and bis the merged bias vector. $x_{t}^{\prime }$ is the input at time t, ${h^{\prime \prime }}_{t}$, ${i^{\prime \prime }}_{t}$, ${o^{\prime \prime }}_{t}$, ${C^{\prime \prime }}_{t}$ are the current memory unit of input gate, output gate, forget gate, and cell state unit, respectively, σ(⋅) is the sigmoid activation function, and tanh(⋅) denotes the hyperbolic tangent function. The memory unit and hidden state through two independent equations are updated:

(39) $$C_{t}=f_{t}\cdot C_{t-1}+i_{t}\cdot {\tilde{C}}_{t}$$

(40) $$h_{t}=o_{t}\cdot \tanh(C_{t})$$

### 4.2. Cross-Modal Attention Fusion Hierarchical Federated Learning

Time attention mechanism: To enhance the interpretability and focus of the model, we introduce an time attention mechanism on top of the LSTM outputs. The time attention mechanism allows the model to dynamically assign importance scores to different time steps, effectively emphasizing the most relevant historical features. For each time step t, an intermediate attention score is computed as:

(41) $$\phi_{t}=\vartheta \tanh\left(w^{\prime }h_{t}^{\prime }+b^{\prime }\right),$$

where $w^{\prime }$ and ϑ are trainable parameter vectors and $b^{\prime }$ is the bias term.

These scores are then normalized using the softmax function to obtain the time attention weights:

(42) $$\partial_{t}=\mathrm{softmax}(\phi_{t})$$

where $\mathrm{softmax}\left(\cdot \right)$ is the normalized exponential function. Finally, the time- and attention-weighted output is computed as a weighted sum of all LSTM hidden states:

(43) $$h^{\mathrm{attn}}=\sum_{t=1}^{T}\partial_{t}h_{t}^{\prime }.$$

This aggregated context vector $h^{\mathrm{attn}}$ is passed to the output layer for final carbon price prediction.

Spatial attention mechanism: Construct a region association matrix through graph neural networks to highlight the contributions of highly correlated nodes. The core idea of graph attention network (GAT) has been adopted. The multi-head attention mechanism calculates the dynamic correlation weights between nodes. Weighted summation aggregates neighbor information and introduces multi-head attention to enhance stability. The spatial attention coefficient of client $n_{client}$ to client $n_{client}^{\prime }$:

(44) $$\begin{array}{c} \partial_{n_{client},n_{client}^{\prime }}=\mathrm{softmax}\left(\mathrm{LeakyReLU}\left(a^{T}\left[w^{\prime \prime }\cdot h_{n_{client}}⊕w^{\prime \prime }\cdot h_{n_{client}^{\prime }}\right]\right)\right) \end{array}$$

where $\mathrm{softmax}\left(\cdot \right)$ is the normalized exponential function, $h_{n_{client}}$ and $h_{n_{client}^{\prime }}$ are the original feature vector of client $n_{client}$ and $n_{client}^{\prime }$, $w^{\prime \prime }$ is the learnable weight matrix used for feature transformation, and $a^{T}$ is the parameter vector of spatial attention mechanism. $\mathrm{LeakyReLU}\left(\cdot \right)$ (Leaky Rectified Linear Unit) is an improved version of the ReLU activation function. ⊕ is a concatenation operation, which means connecting multiple vectors along a specific dimension to form a longer vector.

The objective function of federated learning: The global model parameter is $W_{global}$, the local model parameter of the $n_{client}$ client is $W_{client}$. The objective function of federated learning is:

(45) $$L_{total}=\sum_{n_{client}=1}^{N_{client}}\left[\alpha_{n_{client}}\cdot L_{n_{client}}\left(W_{n_{client}},D_{n_{client}}\right)+\lambda_{g}\cdot {\left\|W_{n_{client}}-W_{global}\right\|}^{2}\right]$$

where, $L_{total}$ is the total loss function of the federated learning system, $N_{client}$ is the total number of clients participating in federated learning, $n_{client}$ is the client index $n_{client}=1,2,\ldots ,N_{client}$, $\alpha_{n_{client}}$ is the weight coefficient of the $n_{client}$-th client, used to balance the importance of different clients, $L_{n_{client}}\left(W_{n_{client}},D_{n_{client}}\right)$ is the loss function of the $n_{client}$-th client on the local dataset $D_{n_{client}}$, $W_{n_{client}}$ is the local model parameter of the $n_{client}$-th client, $D_{n_{client}}$ is the local training dataset of the $n_{client}$-th client, $\lambda_{g}$ is the regularization coefficient, which controls the strength of model aggregation, $W_{global}$ is the globally shared model parameter, and ${\left\|\cdot \right\|}^{2}$ is the Euclidean norm (L2 norm). The steps for hierarchical federated learning are as follows:

- Step 1: The cloud server initializes the global model $W_{\mathrm{global}}$ and regularization coefficient $\lambda_{g}$.
- Step 2: The cloud server selects edge nodes to participate in this round of training according to the data distribution strategy. The total number of edge nodes is denoted as $N_{\mathrm{node}}$.
- Step 3: Each edge node downloads the initial model parameters from the cloud server, including global model weights $W_{\mathrm{global}}$, hyperparameter configurations, and training control parameters.
- Step 4: Each edge node $n_{\mathrm{node}}\in N_{\mathrm{node}}$ synchronously connects to a group of clients.
- Step 5: Each client $n_{\mathrm{client}}\in N_{\mathrm{client}}$ uses its local data to train the received model.
- Step 6: After local training, each client updates its local model parameters $\left(\alpha_{n_{\mathrm{client}}},W_{n_{\mathrm{client}}},D_{n_{\mathrm{client}}}\right)$.
- Step 7: Upon completing training, each client uploads the updated parameters $\left(\alpha_{n_{\mathrm{client}}},W_{n_{\mathrm{client}}},D_{n_{\mathrm{client}}}\right)$ to its corresponding edge node.
- Step 8: Each edge node aggregates all uploaded client parameters (e.g., $\alpha_{n_{\mathrm{client}}}$, $W_{n_{\mathrm{client}}}$, $D_{n_{\mathrm{client}}}$) to form an intermediate edge-level model using incremental aggregation.
- Step 9: Each edge node uploads the aggregated parameters to the cloud server, reducing communication frequency through the edge-level computation.
- Step 10: The cloud server aggregates all model parameters uploaded by the edge nodes (i.e., $\alpha_{n_{\mathrm{client}}}$, $W_{n_{\mathrm{client}}}$, $D_{n_{\mathrm{client}}}$ across all clients and nodes) to update the global parameters $\left(W_{\mathrm{global}},\lambda_{g}\right)$.
- Step 11: The cloud server executes the optimization objective (Equation (45)) and broadcasts the updated global model parameters $\left(W_{\mathrm{global}},\lambda_{g}\right)$ to all edge nodes to start the next training iteration.

## 5. Results and Discussion

This section presents the experimental setup and evaluation results of the proposed model. In the research process of simulating real network environments, Mininet (a process virtualization network simulation tool) is used to construct the network topology structure. Each perception client is mainly composed of local models, IoT sensors, web servers, and data storage units. Each client is responsible for collecting and training. All experiments of perception client were conducted on a Windows 10 operating system using an Intel Core i7-10750H processor. The implementation was carried out using MATLAB (2024b) and Python (version 3.7). It should be noted that the current work demonstrates the conceptual feasibility of a privacy-preserving federated learning framework under a small-scale simulated environment, rather than a real-world deployment involving millions of clients. The scalability and privacy-preserving mechanisms were implemented conceptually within the simulation framework, and full-scale deployment is considered future work. The process of hierarchical federated learning is as follows:

Initialize global model: The server generates initial parameters for the deep learning model.

Client local training: Each client trains a deep learning model based on local data, calculates gradients or updated parameters.

Server aggregation update: The server collects model updates from clients and aggregates them through strategies such as weighted averaging to generate a new global model.

### 5.1. Evaluation Metrics

To assess the model’s effectiveness, standard evaluation metrics are employed. To enhance prediction accuracy, the sequence data $D=\left\{\varphi ({\tilde{X}}_{m,t})⊕\tilde{y}(t)\right\}$ are normalized as follows:

(46) $$D^{\prime }=\frac{D-D_{\min}}{D_{\max}-D_{\min}}$$

where $D^{\prime }$ denotes the normalized data, D is the original data, $D_{\max}$ is the maximum value, and $D_{\min}$ is the minimum value in the original dataset.

The following four metrics are used to quantify the prediction accuracy of the model. The Mean Absolute Error (MAE) is defined as:

(47) $$MAE=\frac{1}{n}\sum_{i=1}^{n}\left|{\hat{y}}_{i}-y_{i}\right|,$$

which measures the average magnitude of the prediction errors. The Mean Absolute Percentage Error (MAPE) is given by:

(48) $$MAPE=\frac{1}{n}\sum_{i=1}^{n}\left|\frac{{\hat{y}}_{i}-y_{i}}{y_{i}}\right|,$$

and provides a relative error in percentage terms. The Root Mean Square Error (RMSE) is calculated as:

(49) $$RMSE=\sqrt{\frac{1}{n}\sum_{i=1}^{n}{\left({\hat{y}}_{i}-y_{i}\right)}^{2}},$$

which penalizes larger errors more heavily and reflects the standard deviation of the prediction residuals. Finally, the Coefficient of Determination ($R^{2}$) is used to evaluate the goodness of fit:

(50) $$R^{2}=1-\frac{\sum_{t=1}^{T}{\left(\hat{y}(t)-y(t)\right)}^{2}}{\sum_{t=1}^{T}{\left(y(t)-\bar{y}\right)}^{2}},$$

where $\bar{y}$ is the mean of the observed values. An $R^{2}$ value close to 1 indicates strong predictive performance. Together, these metrics provide a comprehensive assessment of the model’s prediction accuracy, robustness, and generalization ability.

### 5.2. Effectiveness of Carbon Price Decomposition

The carbon price data were obtained from the Guangzhou Carbon Emissions Exchange, one of China’s pilot carbon trading markets established to test mechanisms ahead of the national ETS launched in 2021. As a major economic center in southern China, Guangzhou provides an important case for examining how regional carbon pricing interacts with national ETS policies. The dataset was partitioned such that approximately 90% of the data was used for training (2015–2023), and the remaining approximately 10% (2024) was reserved for model testing to ensure chronological integrity.

In addition to the target carbon price series, a set of external influencing variables was incorporated to improve forecasting accuracy. These include macroeconomic indices, commodity prices, exchange rates, and international carbon prices. A complete list and discussion of their economic rationale is provided in Section 3.3.

Before model training, the dataset underwent a series of preprocessing steps. Missing and abnormal values were handled through data cleaning routines. For time series with high-frequency noise, such as the EURO STOXX 50 Index and CSI 300 Index, a five-day sliding mean was applied. For volatile commodity prices (e.g., Brent crude oil, NYMEX natural gas), outliers were truncated based on confidence intervals to mitigate extreme fluctuations. All continuous variables, including carbon prices and influencing factors, were standardized using Z-score normalization to ensure numerical comparability and model stability.

**Figure 4:**
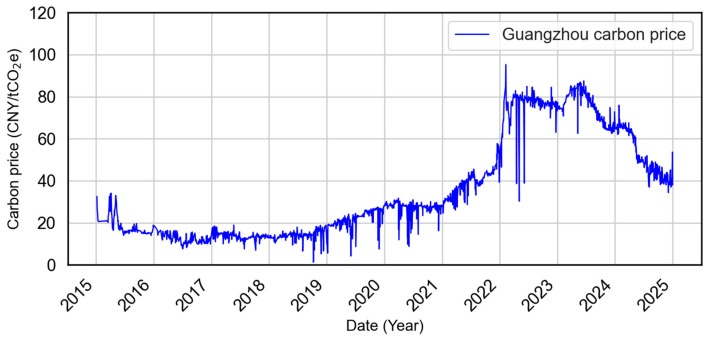
Guangzhou carbon price time series (2015–2024). The market experienced a prolonged low-price phase from 2015 to 2020, a rapid surge in mid-2022 to mid-2023, and a gradual decline thereafter.

As shown in Figure 4, Guangzhou carbon prices exhibit significant temporal dynamics. From 2015 to 2020, prices remained low and stable. Beginning in 2021, they rose steadily, peaking in 2023, followed by a soft decline. This nonstationary behavior, marked by abrupt structural changes, necessitates robust decomposition techniques capable of isolating meaningful patterns across temporal scales.

**Figure 5:**
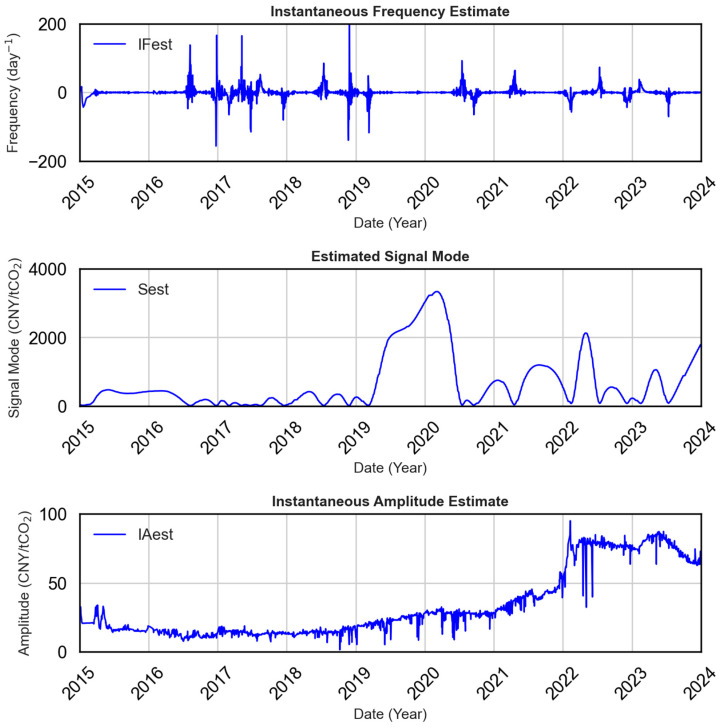
Decomposition results of carbon prices in the Guangzhou market. Top: estimated instantaneous frequency; Middle: decomposed mode components; Bottom: instantaneous amplitude, indicating smoothed price trends.

To address this, the signal is decomposed into subcomponents with distinct instantaneous frequencies and amplitudes, facilitating improved learning from nonstationary data. The decomposition results are shown in Figure 5.

The top panel of Figure 5 shows the instantaneous frequency, capturing periods of sharp fluctuation. The middle panel displays the decomposed mode components, adaptively segmented into broadband or narrowband regions. The framework employs adaptive bandwidth adjustment and spectrum sensing to distinguish meaningful signal components from noise. This enhances the signal-to-noise ratio and reduces the impact of nonstationarity.

In summary, the temporal structure and stability of the carbon price input is improved, laying a stronger foundation for accurate downstream modeling with the model framework.

### 5.3. Impact of Feature Selection and Lag Modeling

#### 5.3.1. Selection and Analysis of Influencing Variables

To ensure a comprehensive and interpretable forecasting model, we first curated a set of market influencing variables based on economic theory and empirical relevance to carbon price fluctuations. These variables reflect macroeconomic activity, energy market dynamics, and financial sentiment. Below is a brief description of the candidate variables:

- EUA Carbon Price: The allowance price in the EU Emissions Trading System, representing international carbon market trends. Data are sourced from www.carbonmonitor.org.cn.
- Steam Coal Price Index: A domestic index reflecting the average cost of thermal coal in China, indicative of power generation costs. Data are sourced from the China Coal Transportation and Distribution (CCTD) website.
- Brent Crude Oil Futures: A benchmark for global crude oil prices, affecting fuel substitution and overall energy market sentiment. Data are sourced from investing.com.
- Europe Stoxx 50 Index: A major European equity index used as a proxy for economic growth and investment sentiment in developed economies. Data are sourced from investing.com.
- CSI 300 Index: Represents the performance of the top 300 A-share stocks in Shanghai and Shenzhen, reflecting domestic financial market conditions. Data are sourced from the China Financial Futures Exchange.
- USD/RMB Exchange Rate: Captures fluctuations in the value of the Chinese yuan relative to the US dollar, impacting import energy prices. Data are sourced from investing.com.
- Euro/RMB Exchange Rate: Represents the currency linkage with the Eurozone, affecting import/export cost structures. Data are sourced from xe.com.
- NYMEX Natural Gas Futures: Reflects international gas price trends, providing insight into fuel substitution and emissions displacement. Data are sourced from investing.com.
- Steam Coal ARA Port Spot Price: The spot price of coal at Amsterdam-Rotterdam-Antwerp, serving as a global benchmark for seaborne thermal coal. Data are sourced from Platts Energy Information.

To quantify the time-lagged influence of market factors on carbon prices, we conduct a lag detection analysis using Granger causality tests, mutual information (MIC), and cross-correlation. These methods help identify the most relevant features not only in terms of magnitude but also in terms of the timing of their impact. Such modeling is crucial for carbon markets, where price dynamics are not only volatile but also reactive to delayed economic signals.

**Figure 6:**
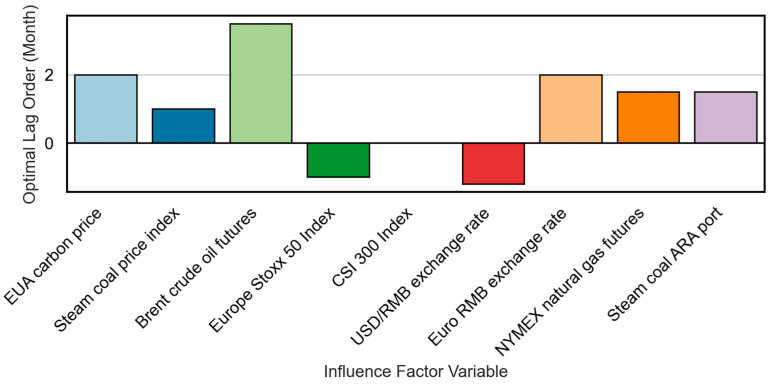
Optimal lag order τ (Month) of major influencing factor variables in the Guangzhou market.

Figure 6 shows the optimal lag order (in months) for each core influencing factor, determined by maximizing the correlation under a significance threshold of p<0.05 from the Granger causality test. This lag structure is used to build a dynamic lag characteristic matrix that captures the temporal dependency and predictive lag pattern for each variable.

**Figure 7:**
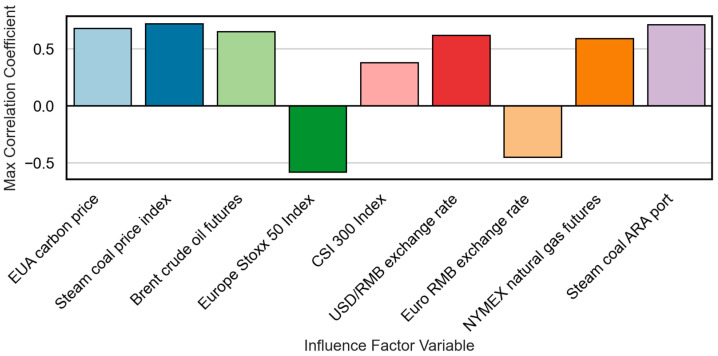
Maximum MIC correlation coefficients between lagged influencing factors and carbon prices.

Figure 7 presents the maximum MIC values obtained from the cross-correlation tests. When the lagged feature $\varphi (x_{t-\tau }^{\left(i\right)})$ and the target $y_{t}^{\left(j\right)}$ are completely independent, the MIC is close to zero. Higher values indicate stronger nonlinear dependencies, highlighting the ability of some variables to explain hidden or complex relationships in the pricing process.

**Figure 8:**
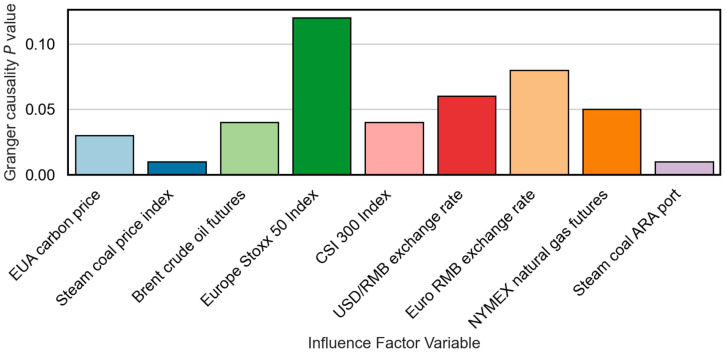
Granger causality p-values of influencing factor variables.

Granger causality values of each factor are shown in Figure 8. A p-value less than 0.05 indicates statistically significant Granger causality. This not only confirms the predictive utility of certain features but also supports their causal precedence in influencing carbon price changes.

The combined results demonstrate that steam coal prices at ARA Port (MIC=0.71, GC=0.01), steam coal index (MIC=0.72, GC=0.01), Brent crude oil (MIC=0.65, GC=0.04), and EU EUA carbon prices (MIC=0.68, GC=0.03) have strong predictive power for Guangzhou carbon prices, with optimal lags ranging from one to two months. Conversely, variables such as the Euro Stoxx 50 Index (MIC=−0.58, GC=0.12) and the euro exchange rate (MIC=−0.45, GC=0.08) exhibit weak or no statistical causality, suggesting indirect or delayed effects through broader macroeconomic channels.

#### 5.3.2. Feature Importance Ranking and Final Selection

Following lag detection, we conducted feature selection and importance evaluation. Bayesian optimization is used to tune hyperparameters. Hyperparameters including maximum tree depth, learning rate, subsampling rate, and column sampling rate are optimized using Bayesian optimization. Table 1 summarizes the optimization results. This grid-based optimization ensures that the model architecture is tailored to the underlying feature structure and avoids overfitting.

**Figure 9:**
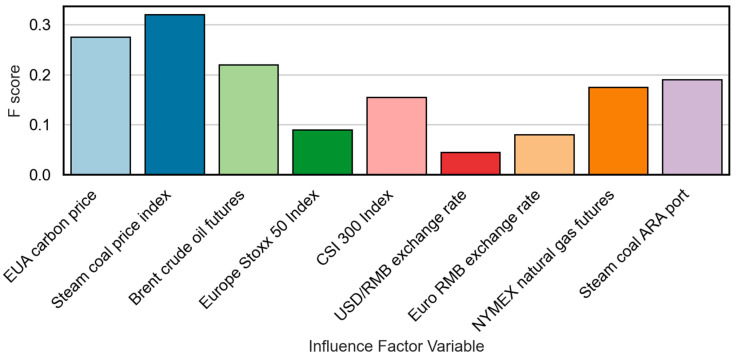
F-score importance of influencing factor features.

Figure 9 illustrates the feature importance scores (F-score) of input variables. These scores provide a quantitative basis for selecting the most relevant features. The F-score reflects how frequently a feature is used in the model’s decision trees and with what discriminative power.

The top features, including EU EUA prices, thermal coal indices, Brent crude oil, and NYMEX natural gas, exhibit both high F-scores and statistically significant Granger causality, supporting their inclusion in the final prediction model. A one to three-month lag feature matrix is constructed to dynamically capture market shifts and short-term policy responses. Core inputs include the EU EUA carbon price (two-month lag), steam coal price (1-month lag), and CSI 300 index (current value), each capturing a different dimension of supply, demand, and investment climate.

Less informative variables such as the Euro Stoxx 50 and euro exchange rate (with p>0.05) are excluded to reduce noise and the risk of overfitting. However, these variables are still monitored externally to detect possible structural breaks or regime shifts, particularly during currency volatility or global economic shocks. This dynamic selection mechanism enhances model interpretability and robustness in forecasting carbon prices in a rapidly evolving market landscape.

Before model training, the dataset underwent standardized preprocessing. Missing values were imputed using linear interpolation for short gaps and mean substitution for longer gaps. Outliers exceeding three standard deviations were capped using a local smoothing filter. High-frequency noise in indices such as EURO STOXX 50 and CSI 300 was suppressed using a five-day moving average. Volatile commodity series (e.g., Brent crude oil, NYMEX natural gas) were truncated based on confidence intervals to mitigate extreme fluctuations. All continuous variables, including carbon prices and influencing factors, were standardized using Z-score normalization to ensure numerical comparability and model stability.

### 5.4. Prediction Accuracy Comparison

#### 5.4.1. Ablation Study: Effect of Model Components

To examine the contribution of each component in the proposed prediction framework, we performed an ablation study by progressively enabling three key modules: (i) feature selection via XGBoost, (ii) temporal modeling using TCN and LSTM, and (iii) an attention mechanism. Specifically: Model-1 is a baseline TCN model trained using the full set of original features, without feature selection or attention. Model-2 incorporates XGBoost-based feature selection and adopts a hybrid TCN-LSTM architecture for time series modeling. The proposed model builds upon Model-2 by inserting an attention layer after the LSTM module to enhance feature weighting and capture long-range temporal dependencies.

**Figure 10:**
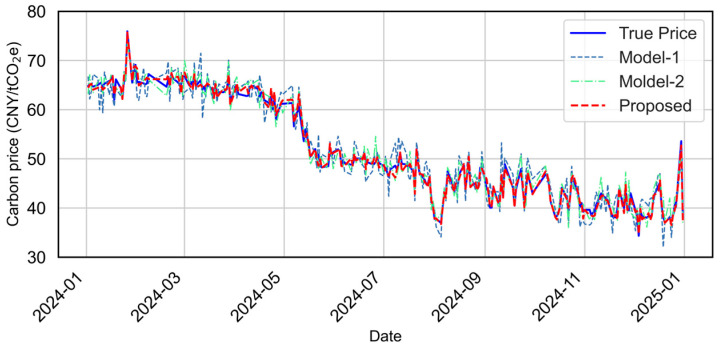
Overall prediction performance of different model configurations for the testing period (2024/01–2024/12), including the TCN (Model-1), feature-enhanced TCN-LSTM (Model-2), and the proposed XGBoost-TCN-LSTM with attention.

**Figure 11:**
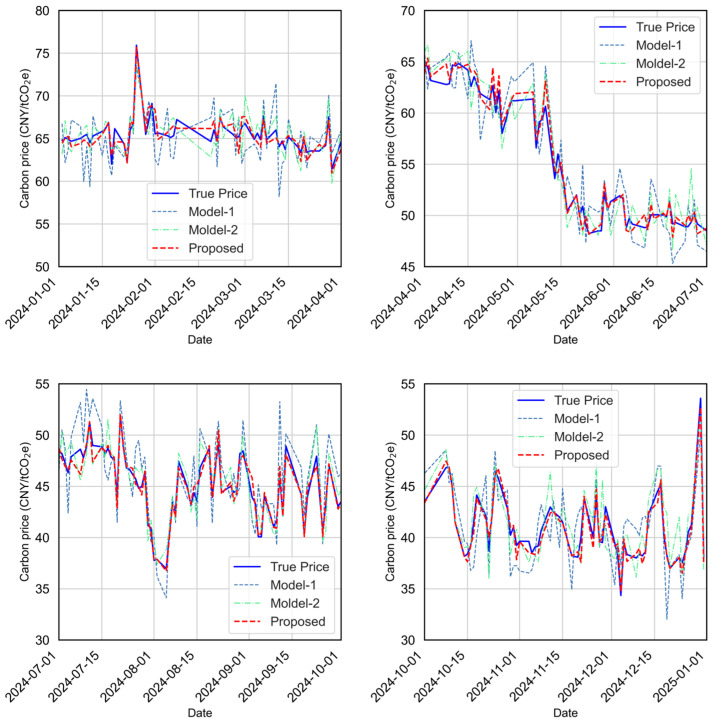
Quarter-wise prediction accuracy comparison for the testing period (2024/01–2024/12) of different model configurations.

**Figure 12:**
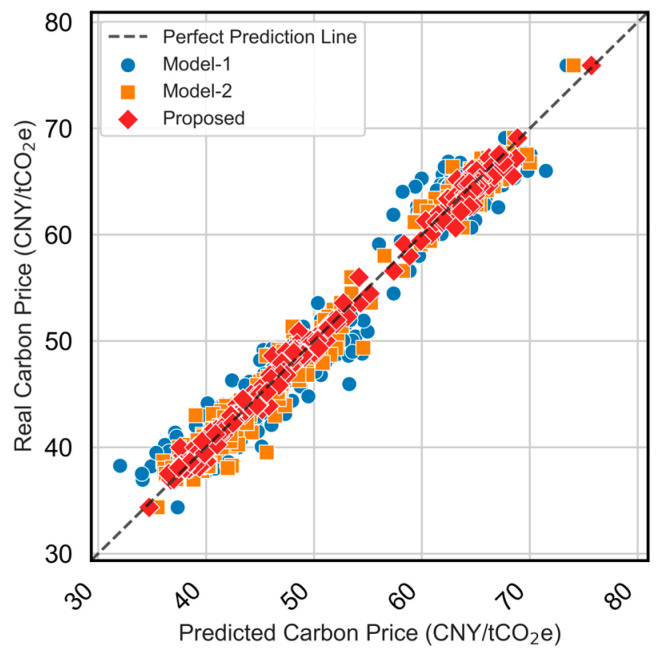
Scatter plot of predicted vs. actual carbon prices for different model configurations for the testing period (2024/01–2024/12).

Model performance is evaluated using RMSE, MAE, MAPE, and R^2^. Overall results are shown in Figure 10, while Figure 11 provides zoomed-in quarterly comparisons to highlight behavior under different market regimes (volatile, stable, rising, and falling periods). Figure 12 presents the predicted-versus-observed scatter plots, and detailed numerical results are summarized in Table 2. The evaluation metrics (RMSE, MAE, and MAPE) are all calculated on the original scale of the carbon price data after reversing the normalization. It should be noted that normalization was applied only during model training to improve numerical stability, and all reported results are converted back to the actual price units for interpretability.

The ablation results reveal several observations. First, XGBoost-based feature selection offers clear benefits, reducing noise in the input space and improving stability across different market phases. Second, combining TCN and LSTM enhances the ability to capture multiscale temporal dependencies, resulting in more accurate and smoother predictions than the baseline. Third, adding attention yields further gains, particularly during periods of sharp market movements, by adaptively emphasizing informative temporal patterns. As visualized in Figure 9, the baseline model exhibits the largest deviation from observations, especially during high-volatility intervals, indicating its sensitivity to redundant features and limited long-term dependency modeling. Model-2 improves markedly in both accuracy and stability, while the proposed model achieves the tightest alignment with ground truth in all quarters and effectively suppresses large-magnitude prediction errors.

Quantitatively, the proposed model attains the best scores across all metrics (e.g., MAE = 0.576, RMSE = 0.772, MAPE = 0.011, R^2^ = 0.994. The scatter in Figure 11 further confirms the strong agreement between predicted and observed values, with the proposed model demonstrating the most compact distribution along the identity line. In summary, the ablation study shows that (i) feature selection is essential for robust carbon price prediction, (ii) the TCN-LSTM combination effectively captures complex temporal dynamics, and (iii) the attention mechanism delivers additional improvements by refining dynamic feature contributions, particularly under volatile market conditions.

#### 5.4.2. Comparison with Baseline Models

To validate the effectiveness of the proposed model framework, we compare it against two representative baseline models: ARMA-GARCH and LSTM-LGBM. ARMA-GARCH is a classical econometric model suitable for modeling volatility in financial time series, while LSTM-LGBM is a hybrid model combining deep sequence learning and gradient boosting trees.

**Figure 13:**
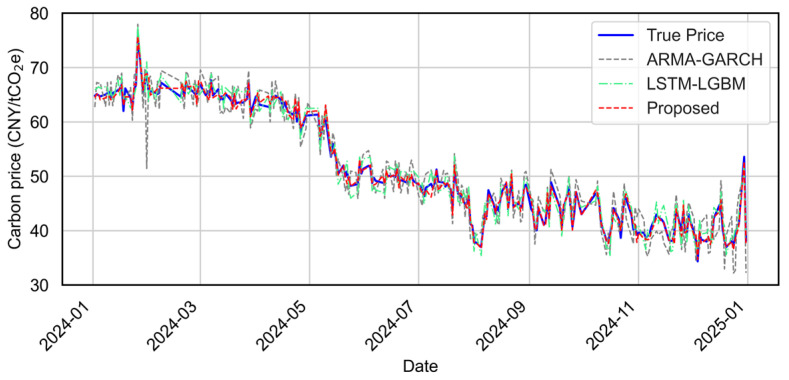
Overall prediction performance comparison among ARMA-GARCH, LSTM-LGBM, and the proposed models for the testing period (2024/01–2024/12).

**Figure 14:**
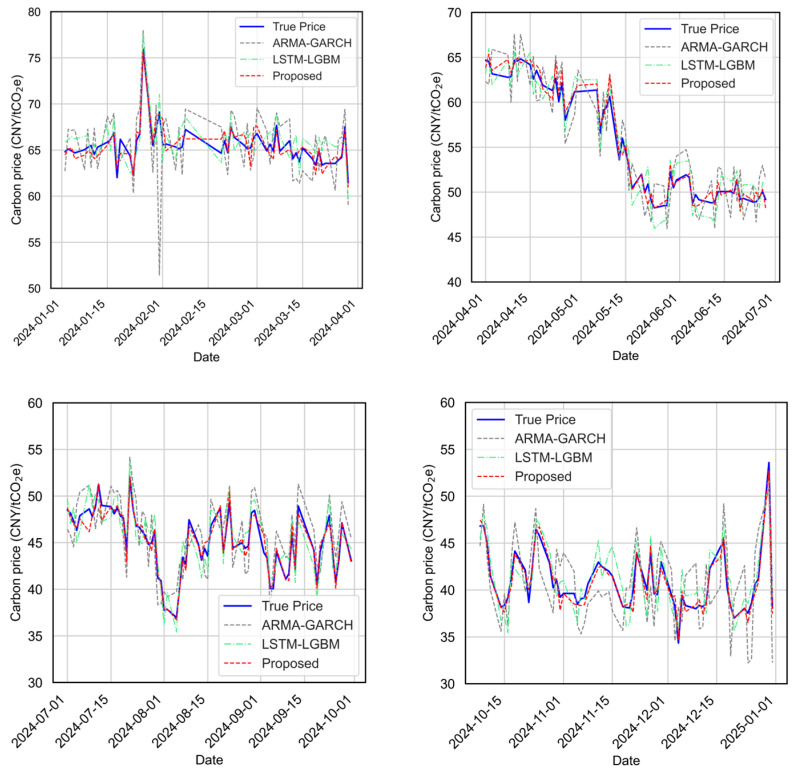
Quarter-wise prediction accuracy comparison across ARMA-GARCH, LSTM-LGBM, and the proposed models for the testing period (2024/01–2024/12).

**Figure 15:**
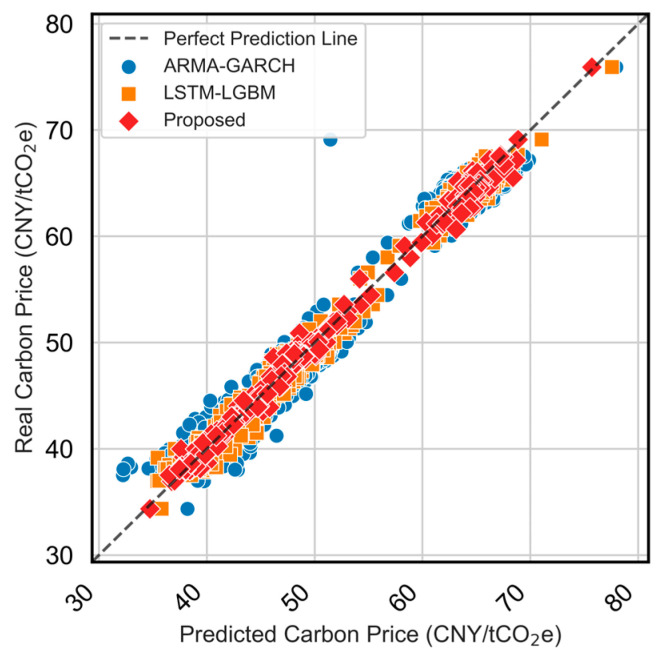
Scatter plot of predicted vs. actual carbon prices for ARMA-GARCH, LSTM-LGBM, and the proposed models for the testing period (2024/01–2024/12).

Figure 13 presents the full-year prediction performance of the three models. Figure 14 further presents results by quarter to illustrate how each model responds to different price dynamics, such as trend shifts and volatility spikes. Figure 15 compares the predicted values versus actual carbon prices for all models. Quantitative evaluation metrics (MAE, MAPE, RMSE, R^2^) are reported in Table 3.

From both the visual and quantitative perspectives, the proposed model framework consistently outperforms the baseline models. As shown in Figure 13, the proposed model predictions closely track the true carbon price trend throughout the year, exhibiting minimal deviation in both smooth and turbulent phases. In quarterly views (Figure 14), the proposed model adapts better to local fluctuations, maintaining higher fidelity across varying temporal patterns.

The scatter plot in Figure 15 further confirms the effectiveness of the proposed model, with its predicted points tightly clustered around the perfect prediction line. Compared to ARMA-GARCH’s underestimation during volatile periods and LSTM-LGBM’s relatively larger amplitude deviations, the proposed model demonstrates more robust performance.

Table 3 shows that the proposed model reduces the MAE and RMSE by more than 50% compared to ARMA-GARCH, and even significantly outperforms the hybrid LSTM-LGBM. The R^2^ of 0.994 indicates near-perfect fitting capability, highlighting the advantages of the proposed model in learning complex temporal dependencies and capturing nonlinear relationships in carbon market dynamics.

After presenting the experimental results, this section further discusses the practical implications of the proposed model. In particular, we highlight how it can support both policy formulation and trading decisions in carbon markets. The proposed model can support both policy formulation and trading decisions in carbon markets. For policy decisions, the model quantifies how policy changes—such as quota adjustments or market expansion—affect carbon prices by tracking real-time correlations between policy variables and market responses. This enables simulation of policy scenarios and evaluation of phased policy impacts. The attention mechanism also helps detect unusual market movements, offering insights into refining regulatory tools like quota reserves. For trading decisions, the model integrates data from multiple regional markets through hierarchical federated learning to generate inter-regional carbon price patterns. These results help identify arbitrage opportunities and volatility risks, while the spatiotemporal attention structure provides early warnings of price shifts, supporting both short-term trading and long-term portfolio planning in the carbon market.

## 6. Conclusions

In order to solve the problem of difficulty in accurately predicting changes in carbon prices due to multiple factors, a perception and prediction of carbon price influencing factors based on hierarchical federated learning and cross-modal spatiotemporal enhanced attention is proposed. This study proposes a novel carbon price prediction model that comprehensively considers the influence of multiple market-driving factors. By applying time–frequency decomposition techniques, the model effectively denoises the carbon price series and enables a more precise analysis of its fluctuation patterns. Dimensionality reduction is employed to extract the most relevant features from high-dimensional influencing factors, while the temporal correlation and hysteresis effects are explicitly captured through lagged modeling strategies. The prediction framework integrates these processed features to forecast carbon prices. Experimental results confirm that the proposed multi-model fusion approach achieves superior predictive performance compared to traditional methods, demonstrating its effectiveness and robustness in modeling the complex dynamics of carbon trading markets. Future work will focus on implementing and experimentally validating hierarchical parameter aggregation mechanisms and secure model fusion strategies for heterogeneous client models within the federated framework. In addition, although the proposed framework simulates a hierarchical federated structure, the present experiments do not involve real confidential data and therefore do not address model weight distillation attacks. Integrating secure aggregation and differential privacy techniques into the hierarchical framework will be an important direction for future work, especially for real industrial deployments with sensitive information.

## Figures and Tables

**Table 1:** Parameter configuration and evaluation results of feature selection model.

Learning Rate	Max Depth	Subsample	Colsample Bytree	MAE	RMSE
0.001	3	0.6	0.6	0.177	0.215
0.001	5	0.8	0.8	0.205	0.253
0.001	7	1.0	1.0	0.191	0.227
0.01	3	0.6	0.6	0.142	0.182
0.01	5	0.8	0.8	0.107	0.121
0.01	7	1.0	1.0	0.157	0.213
0.1	3	0.6	0.6	0.150	0.200
0.1	5	0.8	0.8	0.135	0.197
0.1	7	1.0	1.0	0.159	0.214

**Table 2:** Quantitative performance comparison of different model configurations using MAE, MAPE, RMSE, and R^2^ metrics.

Model	MAE	MAPE	RMSE	R^2^
Model-1 Model-2	2.284 1.357	0.046 0.028	2.571 1.622	0.934 0.975
Proposed	0.576	0.011	0.772	0.994

**Table 3:** Quantitative performance comparison of ARMA-GARCH, LSTM-LGBM, and the proposed models using MAE, MAPE, RMSE, and R^2^ metrics.

Model	MAE	MAPE	RMSE	R2
ARMA-GARCH LSTM-LGBM	2.645 1.513	0.054 0.031	2.911 1.568	0.918 0.976
Proposed	0.576	0.011	0.772	0.994

## Data Availability

Data is available from the corresponding authors upon request.

## Abbreviations

The following abbreviations are used in this manuscript:

ACMD	Adaptive Chirp Mode Decomposition
ARMA	Autoregressive Moving Average
BO	Bayesian Optimization
EEMD	Ensemble Empirical Mode Decomposition
GARCH	Generalized Autoregressive Conditional Heteroskedasticity
KPCA	Kernel Principal Component Analysis
LGBM	Light Gradient Boosting Machine
LSTM	Long Short-Term Memory
MAE	Mean Absolute Error
MAPE	Mean Absolute Percentage Error
RMSE	Root Mean Square Error
RBF	Radial Basis Function
SSA	Sparrow Search Algorithm
TCN	Temporal Convolutional Network
VAR-VEC	Vector Autoregression–Vector Error Correction
VMD	Variational Mode Decomposition
XGBoost	Extreme Gradient Boosting
